# Therapeutic effects of a single injection of human umbilical mesenchymal stem cells on acute and chronic colitis in mice

**DOI:** 10.1038/s41598-019-41910-x

**Published:** 2019-04-09

**Authors:** Yu-Lung Chang, Huei-Yu Lo, Shun-Ping Cheng, Kuo-Ting Chang, Xiu-Fang Lin, Sheng-Ping Lee, Ming-Fa Hsieh, Chin-Kan Chan

**Affiliations:** 1 Department of Urology, Taoyuan General Hospital, Ministry of Health and Welfare, Taoyuan, Taiwan; 2 Department of Biomedical Engineering, College of Engineering, Chung Yuan Christian University, Chung Li, Taiwan; 3 Department of Urology, School of Medicine, National Yang-Ming University, Taipei, Taiwan; 4 Department of Rehabilitation, Taoyuan General Hospital, Ministry of Health and Welfare, Taoyuan, Taiwan; 5 Department of Pediatrics, Taoyuan General Hospital, Ministry of Health and Welfare, Taoyuan, Taiwan; 6 Department of Biotechnology, School of Health Technology, Ming Chuan University, Taoyuan, Taiwan; 7 Translational Medicine Center, Taoyuan General Hospital, Ministry of Health and Welfare, Taoyuan, Taiwan; 8 Department of Chemistry, Chung Yuan Christian University, Chung Li, Taiwan

## Abstract

Multiple injections of bone marrow mesenchymal stem cells (BMMSCs) have been used for treatment of chronic colitis in mice. We aimed to report the therapeutic effects of a single injection of human umbilical cord mesenchymal stem cells (hUCMSCs) on acute and chronic colitis. Male C57BL/6JNarl mice were divided into control, phosphate-buffered saline (PBS), and hUCMSCs treated groups, respectively. Acute and chronic colitis were induced in the mice (except controls) using 3% dextran sulfate sodium (DSS). The mice in the hUCMSCs group underwent a single injection of hUCMSCs. The disease activity index (DAI), colon length, histology, colon inflammation score, *in vivo* stem cells images, and blood cytokine levels were recorded. The DAI was significantly higher in the hUCMSCs group than in the control group and lower than in the PBS group on all days. The colon length was significantly longer and the colon inflammation score was significantly lower in the hUCMSCs group than in the PBS group on days 8 and 25. IL17A, Gro-α, MIP-1α, MIP-2, and eotaxin were significantly lower in the hUCMSCs group than in the PBS group on days 8 and 25. Single-injection hUCMSCs improved DSS–induced acute colitis and decreased progression of acute colitis to chronic colitis.

## Introduction

Ulcerative colitis (UC) is a chronic inflammatory bowel disease (IBD) associated with inflammation of the colon mucosa. UC involves the rectum and may affect part of the colon or the entire colon in an uninterrupted pattern^1^. Although the precise etiology of IBD remains unclear, it is thought that interactions among genetic factors, the host immune system, and environmental factors play important roles in the pathogenesis of IBD^2^. The prevalence of UC is approximately 10–200 cases per 100,000 individuals in North America and Europe. The incidence of UC varies depending on geography, and it is most common in northern Europe and North America. It continues to rise in southern Europe, Asia, and much of the developing world^3^. IBD predominantly affect younger patients of reproductive age, and the age of onset of IBD peaks between 15 and 25 years old^4,5^. The literature has revealed that UC is associated with a variety of extra-intestinal manifestations such as joint, skin, ocular, and oral manifestations, osteoporosis, hepatobiliary disease, and amyloidosis^6–8^. The outcome of medical therapy varies in the ameliorating major symptoms of the disease, in treating extra-intestinal manifestations, and in preventing complications^9^. For those patients suffering the failure of medical treatments, bowel resection is needed, and there is an unmet demand for new therapeutic approaches targeting uncontrollable inflammatory activity^10,11^.

Mesenchymal stem cells (MSCs) are nonhematopoietic multipotent stem cells that can differentiate into adipocytes, chondrocytes, osteocytes, smooth muscle cells, fibroblasts, and hematopoietic supportive stroma and are self-renewable^12–14^. In addition to their ability to promote tissue regeneration from damaged tissue progenitors^15,16^, MSCs have been shown to regulate both innate and adaptive immune responses by inhibiting T cell proliferation, B cell function, and dendritic cell maturation^9,16^. Therefore, MSCs are emerging as a candidate for treatment of immune-mediated disease, including IBD. Human umbilical cord mesenchymal stem cells (hUCMSCs) are isolated from the human umbilical cord. Compared with stem cells from other sources they have inherent functionalities including low immunogenicity, noninvasive harvesting, easy expansion *in vitro*, greater anti-inflammatory effect than bone marrow mesenchymal stem cells (BMMSCs) and ethical access^17,18^. Additionally, the anti-inflammatory and immunomodulatory properties of hUCMSCs make them appealing candidates for cell-based therapies^19^.

Several kinds of MSCs including BMMSCs, hUCMSCs, and human umbilical cord blood-derived MSCs have been proven to reduce colitis in mice^20–22^. In previous studies of colitis in mice, hUCMSCs efficiently colonized the inflamed colon and survived where they directly suppressed the inflammatory and immune responses^22,23^. Most experiments have focused on the improvement of inflammation shortly after the injection of MSCs, and the underlying mechanisms for the beneficial effects of MSCs are not yet fully understood. Recently, a study of the long-term effects of repeated BMMSCs injections in mice with chronic colitis induced by repeated administration of DSS showed that repeated systemic infusion of BMMSCs at the onset of the disease exerted preventive and rapid recovery effects, with long-term immunosuppressive action in mice^11^. However, the therapeutic effects of a single injection of hUCMSCs in the mice bearing DSS-induced acute and chronic colitis have not been disclosed. In literature, BALB/c mice developed an acute form of colitis when exposed to DSS and recover spontaneously about 4 weeks after DSS removal, whereas the C57BL/6 mouse strain has been shown to develop acute colitis, which later progresses to chronic inflammation^24^. As such, we used the C57BL/6 mouse strain to investigate the treatment effects of a single injection of hUCMSCs on one cycle of DSS-induced acute and chronic colitis in mice.

## Results

### Disease activity index (DAI)

**Figure 1:**
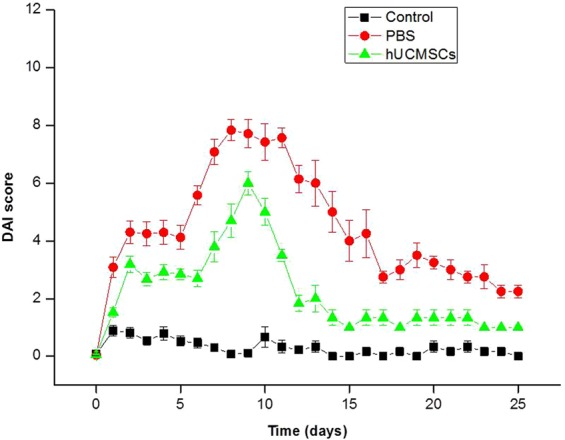
Disease activity index (DAI). The DAI score includes scales for stool consistency, body weight loss, fur texture, and animal posture. The DAI (mean ± SEM) was higher in the PBS group and the hUCMSCs group than in the healthy control group throughout the 25 days of observation with significantly difference (Supplementary Tables S1 and S2) (P < 0.05). The DAI was lower in the hUCMSCs group than in the PBS group throughout the 25 days of observation with significantly difference (Supplementary Table S3) (P < 0.05).

Figure 1 shows DAI data for mice of control, PBS and hUCMSCs groups. The DAIs for hUCMSCs and PBS groups were significantly higher than that in the control group throughout the observation period of 25 days (Supplementary Table S1 and S2) (P < 0.05). Meanwhile, The DAI was significantly lower in the hUCMSCs group than that in the PBS group throughout the observation period (Supplementary Table S3) (P < 0.05).

### Colon length

**Figure 2:**
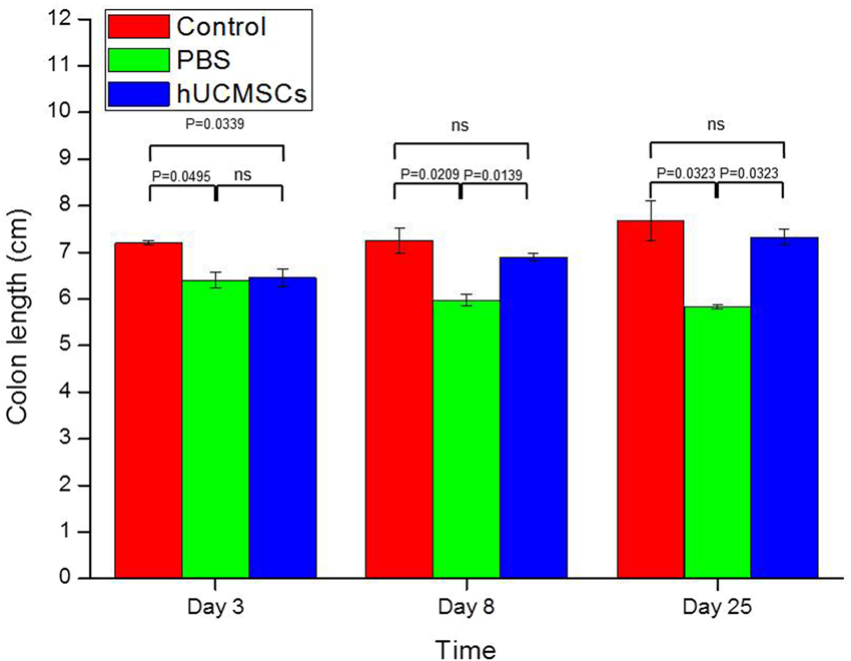
Colon length. The mean (±SEM) colon length was longer in the hUCMSCs group than that in the PBS group on days 8 (P = 0.0139) and 25 (P = 0.0139). The colon length was shorter in the hUCMSCs group than that in the control group on days 8 (P = 0.5316) and 25 (P = 0.3094) but without significantly difference. ns: not significant.

The data of colon length of mice is shown in Fig. 2. The colon length of the mice on day 3 was 7.20 ± 0.05 cm in the control group, 6.40 ± 0.17 cm in the PBS group, and 6.45 ± 0.18 cm in the hUCMSCs group. The colon length on day 8 was 7.25 ± 0.26 cm in the control group, 5.98 ± 0.13 cm in the PBS group, and 6.90 ± 0.07 cm in the hUCMSCs group. The colon length on day 25 was 7.68 ± 0.42 cm in the control group, 5.83 ± 0.05 cm in the PBS group, and 7.33 ± 0.17 cm in the hUCMSCs group. The colon length was longer in the hUCMSCs group than that in the PBS group on days 8 (P = 0.0139) and 25 (P = 0.0139). The colon length was shorter in the hUCMSCs group than that in the control group on days 8 (P = 0.5316) and 25 (P = 0.3094) but without significantly difference.

### Colon Histology

**Figure 3:**
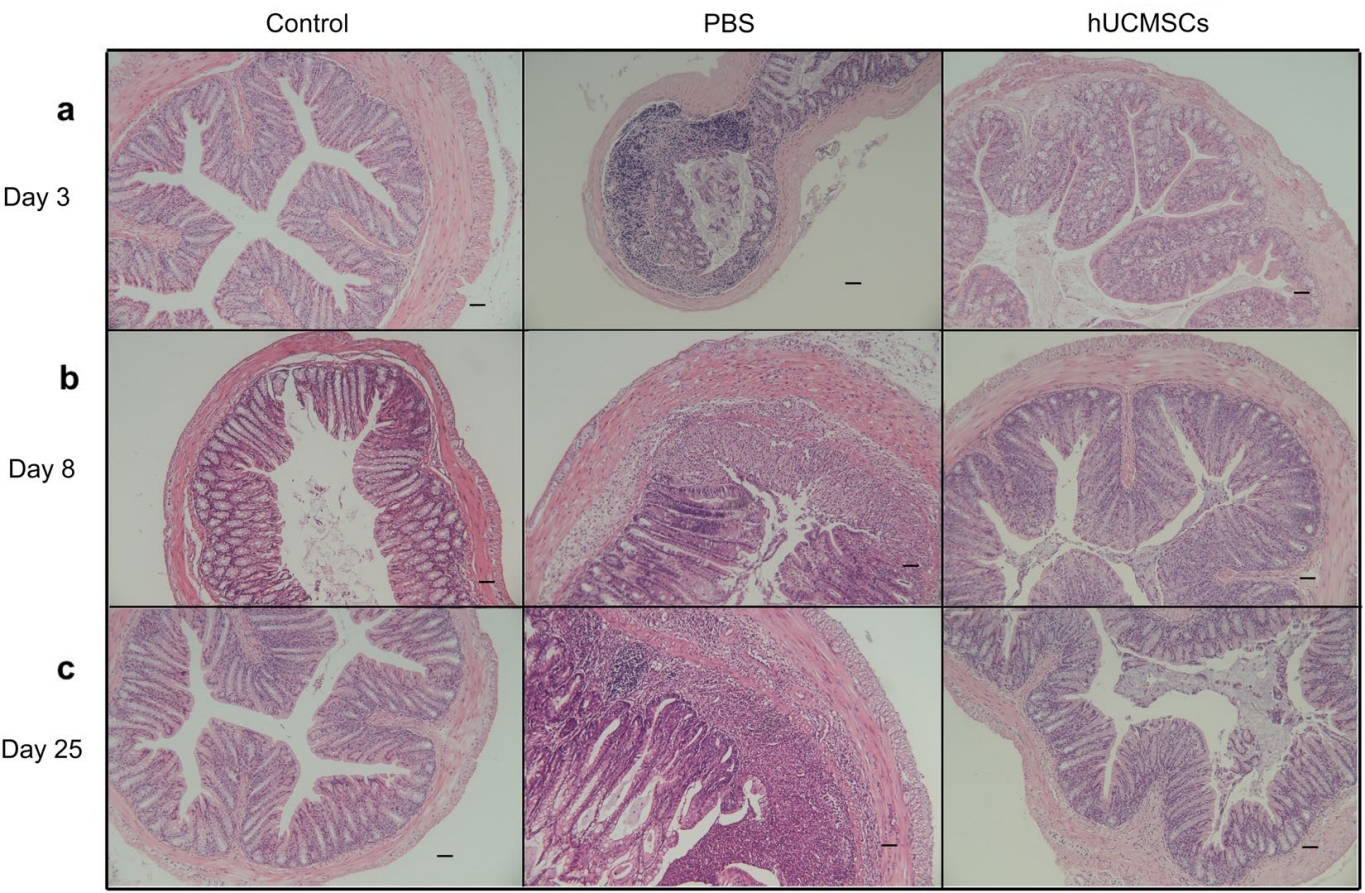
Colon histology. In the healthy control group, the colon architecture was normal, without inflammatory cell infiltrates. In the PBS group, epithelial erosion to extensive ulceration was noted, and submucosal to transmural inflammatory cell infiltration was observed. In the hUCMSCs group, milder epithelial erosion and inflammatory cell infiltration were noted than in the PBS group. Sections were stained with hematoxylin and eosin. Original magnification x100. Scale bar is 50 µm.

**Figure 4:**
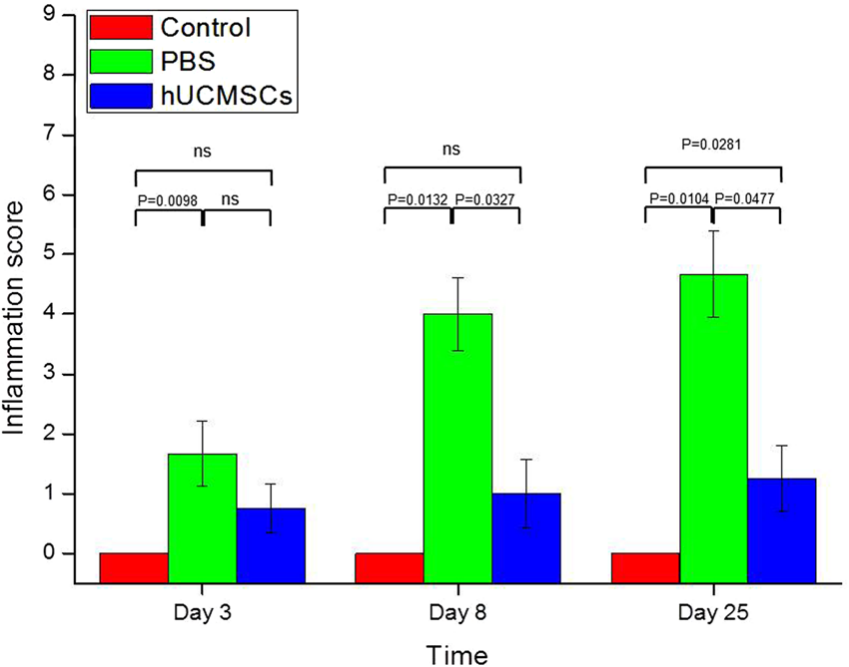
Colon inflammation score. The mean (±SEM) inflammation score was lower in the hUCMSCs group than in the PBS group on days 8 (P = 0.0327) and 25 (P = 0.0477). ns: not significant.

The colon histology of the mice is shown in Fig. 3, and the inflammation score of colon is shown in Fig. 4, respectively. The inflammation score on day 3 was 0.0 ± 0.0 in the healthy control group, 1.7 ± 0.5 in the PBS group, and 0.8 ± 0.4 in the hUCMSCs group. The inflammation score on day 8 was 0.0 ± 0.0 in the control group, 4.0 ± 0.6 in the PBS group, and 1.0 ± 0.6 in the hUCMSCs group. The inflammation score on day 25 was 0.0 ± 0.0 in the control group, 4.7 ± 0.7 in the PBS group, and 1.3 ± 0.5 in the hUCMSCs group. The inflammation score was significantly lower in the hUCMSCs group than in the PBS group on days 8(P = 0.0327) and 25 (P = 0.0477).

### ***In vivo*** imaging of stem cells stained with near-infrared (NIR) dyes

**Figure 5:**
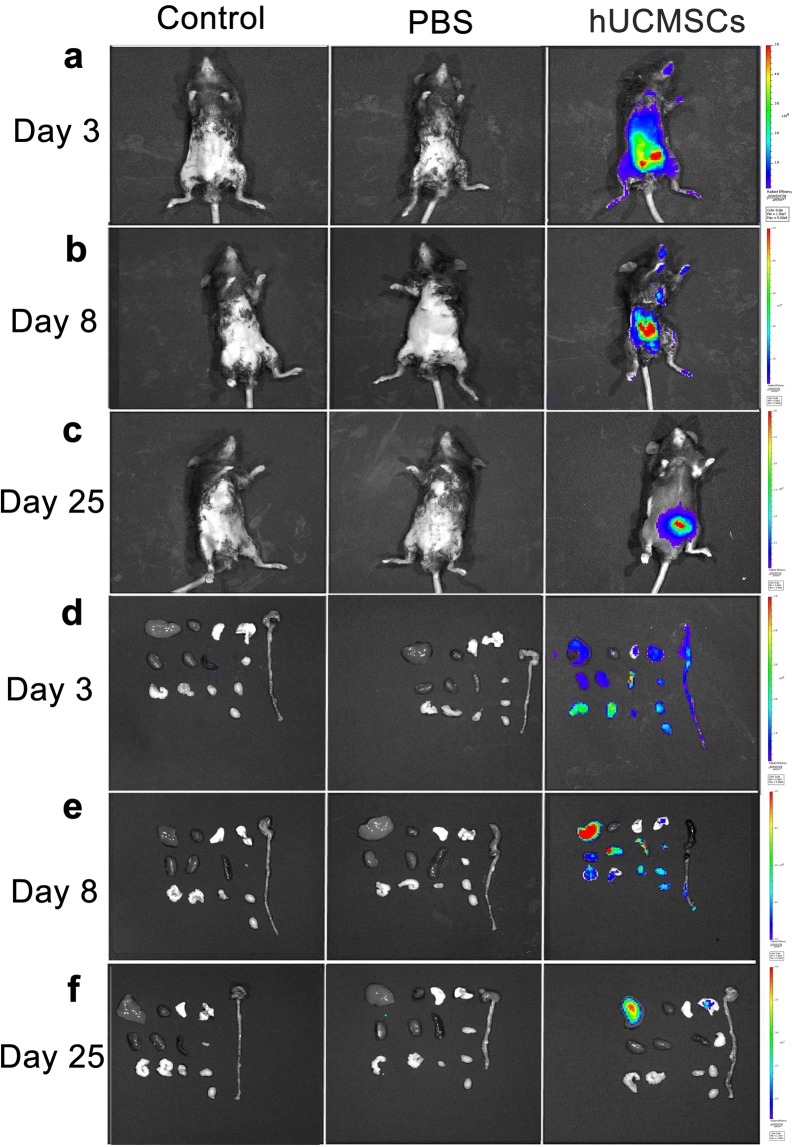
*In vivo* imaging. hUCMSCs were labeled with DiR dye. In the whole-body images, hUCMSCs survived at least 25 days (**a**–**c**). On days 3 and 8, the hUCMSCs could be seen in the lungs, liver, spleen, kidneys, colon, heart, mesenteric lymph nodes, testes, seminal vesicles, and bladder (**d**,**e**). On day 25, the hUCMSCs could only be seen in the liver and lungs (**f**).

The *in vivo* images of the whole-body and the major organs of the mice are shown in Fig. 5. In the whole-body images, hUCMSCs survived at least 25 days post injection. On days 3 and 8, the stem cells could be seen in the lungs, liver, spleen, kidneys, colon, heart, mesenteric lymph nodes, testes, seminal vesicles, and bladder, respectively. On day 25, the stem cells could be seen only in liver and lung. Besides, the stem cells were also confirmed by the immunohistochemistry staining (Supplementary Figs S1 to S3).

### Cytokine and chemokine levels

**Figure 6:**
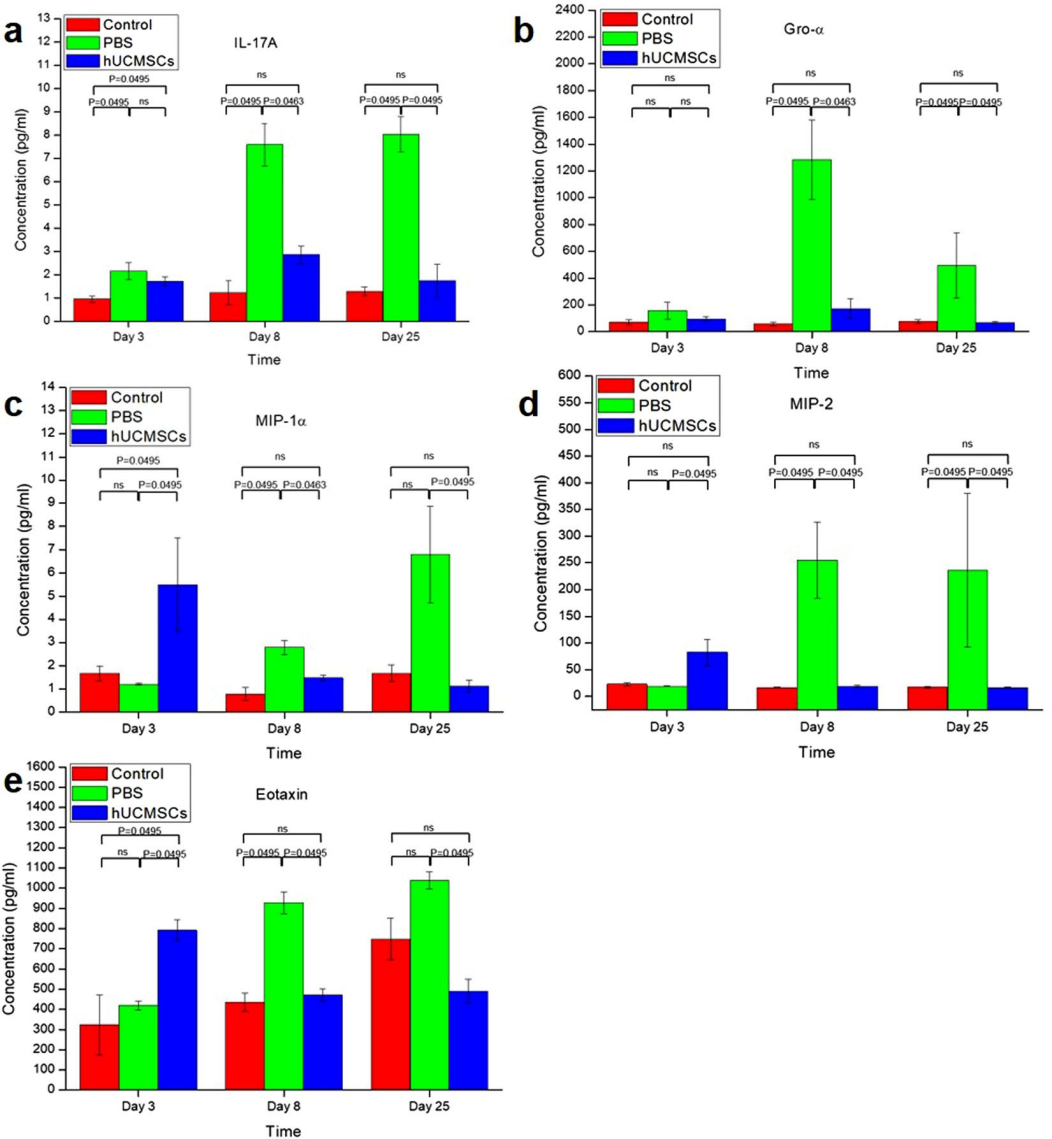
Mean (±SEM) serum cytokine and chemokine levels. MIP-1α, MIP-2, and eotaxin were higher in the hUCMSCs group than the PBS group at day 3 (P = 0.0495, P = 0.0495 and P = 0.0495 respectively). IL17A, Gro-α, MIP-1α, MIP-2, and eotaxin were lower in the hUCMSCs group than the PBS group at day 8 (P = 0.0463, P = 0.0463, P = 0.0463, P = 0.0495 and P = 0.0495 respectively) and 25 (all P = 0.0495). ns: not significant.

The cytokine and chemokine levels are summarized in Fig. 6. IL17A at day 3 was 0.95 ± 0.13 pg/mL in the control group, 2.15 ± 0.38 pg/mL in the PBS group, and 1.72 ± 0.20 pg/mL in the hUCMSCs group. IL17A at day 8 was 1.23 ± 0.51 pg/mL in the control group, 7.59 ± 0.91 pg/mL in the PBS group, and 2.86 ± 0.36 pg/mL in the hUCMSCs group. IL17A at day 25 was 1.28 ± 0.19 pg/mL in the control group, 8.05 ± 0.75 pg/mL in the PBS group, and 1.73 ± 0.73 pg/mL in the hUCMSCs group.

Gro-α at day 3 was 71.30 ± 20.38 pg/mL in the control group, 156.47 ± 63.82 pg/mL in the PBS group, and 95.62 ± 16.76 pg/mL in the hUCMSCs group. Gro-α at day 8 was 59.11 ± 13.11 pg/mL in the control group, 1283.45 ± 298.68 pg/mL in the PBS group, and 172.74 ± 75.08 pg/mL in the hUCMSCs group. Gro-α at day 25 was 77.44 ± 12.42 pg/mL in the control group, 494.83 ± 241.87 pg/mL in the PBS group, and 67.48 ± 7.37 pg/mL in the hUCMSCs group.

MIP-1α at day 3 was 1.66 ± 0.32 pg/mL in the control group, 1.19 ± 0.04 pg/mL in the PBS group, and 5.48 ± 2.02 pg/mL in the hUCMSCs group. MIP-1α at day 8 was 0.78 ± 0.27 pg/mL in the control group, 2.79 ± 0.30 pg/mL in the PBS group, and 1.47 ± 0.12 pg/mL in the hUCMSCs group. MIP-1α at day 25 was 1.67 ± 0.36 pg/mL in the control group, 6.79 ± 2.09 pg/mL in the PBS group, and 1.12 ± 0.25 pg/mL in the hUCMSCs group.

MIP-2 at day 3 was 22.70 ± 3.16 pg/mL in the control group, 19.06 ± 0.97 pg/mL in the PBS group, and 82.84 ± 23.82 pg/mL in the hUCMSCs group. MIP-2 at day 8 was 16.66 ± 0.67 pg/mL in the control group, 255.02 ± 70.82 pg/mL in the PBS group, and 18.94 ± 1.93 pg/mL in the hUCMSCs group. MIP-2 at day 25 was 17.30 ± 1.75 pg/mL in the control group, 236.26 ± 144.02 pg/mL in the PBS group, and 16.40 ± 1.31 pg/mL in the hUCMSCs group.

Eotaxin at day 3 was 323.43 ± 148.62 pg/mL in the control group, 419.27 ± 23.25 pg/mL in the PBS group, and 791.98 ± 50.88 pg/mL in the hUCMSCs group. Eotaxin at day 8 was 435.42 ± 45.22 pg/mL in the control group, 927.85 ± 53.27 pg/mL in the PBS group, and 472.02 ± 29.70 pg/mL in the hUCMSCs group. Eotaxin at day 25 was 748.65 ± 103.54 pg/mL in the control group, 1039.27 ± 41.86 pg/mL in the PBS group, and 490.40 ± 57.58 pg/mL in the hUCMSCs group.

MIP-1α, MIP-2, and eotaxin were higher in the hUCMSCs group than the PBS group at day 3 (P = 0.0495, P = 0.0495 and P = 0.0495 respectively). IL17A, Gro-α, MIP-1α, MIP-2, and eotaxin were lower in the hUCMSCs group than the PBS group at day 8(P = 0.0463, P = 0.0463, P = 0.0463, P = 0.0495 and P = 0.0495 respectively) and 25 (all P = 0.0495). No significant changes in serum concentrations of IFN-γ, IL-12p70, IL-13, IL-1β, IL-2, IL-4, IL-5, IL-6, TNF-α, IL-18, IL-10, IL-22, IL-23, IL-27, IL-9, IP-10, MCP-1, MCP-3, MIP-1β, RANTES, and GM-CSF were detected.

## Discussion

A single injection of BMMSCs and hUCMSCs reduced the severity of acute colitis induced by DSS in the mice^22,25,26^. BMMSCs or hUCMSCs can decrease the severity of acute or chronic colitis induced by DSS or trinitrobenzene sulfonic acid (TNBS) with repeated injections^11,23,27–29^. However, studies of a single injection of hUCMSCs in one cycle DSS-induced acute and chronic colitis are lacking. This study is the first to demonstrate the therapeutic effects of a single injection of hUCMSCs on one cycle of DSS-induced acute and chronic colitis. Secondly, hUCMSCs not only improved DSS-induced acute colitis but also decreased the acute colitis progressing to chronic colitis.

We also confirmed that DSS induced acute and chronic colitis successfully, as shown in the literature^24,26^. The DAI, colon length and histology, and the inflammatory scores for the colon, cytokines, and chemokines (Figs 1–4 and 6) all indicated^24,30^ that a single injection with hUCMSCs improved the severity of acute and chronic colitis better than PBS did (Figs 1–4 and 6). We demonstrated that a single injection of hUCMSCs reduced acute and chronic IBD in the mouse model. The hUCMSCs decreased the progression of acute colitis to chronic colitis. For clinical application, a single injection of stem cells is more convenient than are multiple injections.

The colon histology (Fig. 3) in PBS group shows epithelial erosion to extensive ulceration and inflammatory cell infiltration. One hUCMSCs injection reduced the damage to colon tissue and decreased the inflammatory cell infiltration in the colon tissue. This finding is compatible with previous *in vitro* studies showing that hUCMSCs have immunosuppressive effects on peripheral blood mononuclear cell proliferation^18^. MSCs can regulate both innate and adaptive immune responses by inhibiting T cell proliferation, B cell function, and dendritic cell maturation^9,16,31^.

We found that hUCMSCs decreased serum cytokine IL-17A and chemokines Gro α, MIP-1α, MIP-2, and eotaxin in comparison to PBS in acute colitis (day 8) and chronic colitis (day 25). On day 3, MIP-1α, MIP-2, and eotaxin were higher in the hUCMSCs group than in the PBS group. According to the whole-body hUCMSCs labeling and tracking study, the stem cells can survive in many organs for at least up to 8 days (Fig. 5). However, by day 25, the stem cells could only be found in the liver and lungs. DSS majorly causes disruption of the epithelial layer of the gut, followed by an acute inflammatory response marked by massive infiltration of neutrophils and macrophages into the mucosa^32^ with secretion of many cytokines and chemokines including IL17A, Gro α, MIP-1α, MIP-2, and eotaxin^33–37^. Initially on the day 3, inflammatory cells and hUCMSCs were recruited by chemokines and relatively hUCMSCs may induce transient inflammation. Mouse bone marrow–derived clonal MSCs could show anti-inflammatory effects through direct influence on the inflammatory cells *in vivo*^27^. Khalil *et al*. showed that stem cells were detected predominately in the submucosa of the damaged colonic epithelium and accelerated tissue repair by enhancing microcirculation^38^. According to the literature and our current and previous findings, the possible mechanisms of hUCMSCs to reduce acute colitis and to decrease the progression of acute to chronic colitis may be that initially, these cells migrate to many organs including the colon damaged by DSS induce systemic inflammation response that may then lead to an immunosuppressed status within a few days via feedback mechanisms^39^. Additionally, more hUCMSCs were recruited by chemokines^40,41^ that then helped to repair the epithelial layer of the gut (Fig. 3), decreased the secretion of chemokines by epithelial cells and inflammatory cells, and could have a direct influence on inflammatory cell proliferation^22,40,42^. When the chemokine and cytokine secretion was decreased (Fig. 6), the quantity of recruited inflammatory cells decreased, and the severity of colitis was alleviated. It reflects that serum chemokine concentration is a cumulative level of systemic inflammation, however, the colon accounts for a large proportion. It also indicates a relative site-specific migration pattern of hUCMSCs.

According to the whole-body hUCMSCs labeling and tracking study, the stem cells can survive in many organs for at least up to 8 days (Fig. 5). However, by day 25, the stem cells could only be found in the liver and lungs. Besides, the stem cells could survive up to day 25 were also confirmed by the immunohistochemistry staining (Supplementary Figs S1 to S3). As we known, the stem cells decay over time, there are still some stem cells seen over liver and lung because of the first pass effect and capture effect^43^. When the acute colitis was initially suppressed by the stem cells, the portion of acute colitis progressing to chronic colitis decreased even though only few stem cells survive to day 25. This mechanism needs further investigation to determine and verify the details.

In a model with repeated DSS-induced chronic colitis, 3 systemic infusions of BMMSCs (1 × 10^7^ cells/200 μL) at the onset of the disease exerted preventive and rapid recovery effects, with long-term immunosuppressive action^11^. We found that one injection of hUCMSCs with fewer cells (2 × 10^6^ cells/200 μL) also decreased DSS-induced acute colitis and decreased the progression of acute colitis to chronic colitis. One possible reason for our result is that our previous study indicated that the anti-inflammation effect of hUCMSCs is better than that of BMMSCs^18^. In a comparison of different MSCs delivery routes, the intraperitoneal injection route showed the best colitis recovery and could be the optimum MSCs delivery route for the treatment of DSS-induced colitis^43^. Intraperitoneal injection of hUCMSCs could be a secondary reason that a single injection of hUCMSCs decreased the acute colitis and the progressing to chronic colitis. Intraperitoneal injection is not without risks. Intra-abdominal organ injury and intra-abdominal infection are of significant concern. With the development for IP injection in the field of stem cell therapy, new techniques and designs for reducing such complications are a future direction of study.

## Conclusion

To our best knowledge, we are the first to demonstrate the therapeutic effects of a single injection of hUCMSCs on one cycle of DSS-induced acute and chronic colitis. We found that hUCMSCs not only improved DSS-induced acute colitis but also decreased the acute colitis progressing to chronic colitis. Further research is needed to elucidate the mechanisms of action, minimum effective dosing, and optimum route of administration. Clinical studies in patients are a future requirement.

## Methods

### Ethics statement

This work was approved by the institutional review board of Taoyuan General Hospital, Ministry of Health and Welfare, Taoyuan, Taiwan (IRB Number: TYGH104043). All women provided written informed consent. The research was conducted in accordance with Helsinki Declaration. All animal experiments were performed in accordance with relevant guidelines and regulations of Animal Ethics Committee of Chung Yuan Christian University (IACUC Approval Number: 105013) accredited for laboratory animal care by Ministry of Health and Welfare of Taiwan, Republic of China.

### hUCMSCs culture

The hUCMSCs were derived from the Wharton’s jelly of human umbilical cords were cultured in a flask with minimum essential medium α (Invitrogen, Life Technologies Corporation, Gaithersburg, MD) containing 5% UltraGRO (Helios Bioscience, AventaCell BioMedical Corporation) and 1% penicillin-streptomycin. TrypLE (Gibco, Life Technologies Corporation) was used to harvest the cells. Oil red O stain and von Kossa stain were used to verify that the cells preserved their differentiation capacity after long-term maintenance in cell culture^18,42^.

### DSS-induced colitis model and hUCMSCs transplantation

Male C57BL/6JNarl mice (aged 6–8 weeks) were purchased from the National Laboratory Animal Center (Taipei, Taiwan). All mice were housed in a temperature and humidity-controlled room and were allowed free access to an *in vivo* imaging diet (Caliper Life Sciences, Hopkinton, MA) for 2 weeks before the imaging study. The mice were sacrificed by cervical dislocation.

The mice in the PBS and hUCMSCs groups were given 3% DSS (45 kDa; TdB Consultancy, Uppsala, Sweden) ad libitum in their drinking water for 6 days followed by water for the remainder of the study. The stages of colitis were defined as follows: days 3 and 8 as early colitis (characterized by considerable neutrophil influx into the colon), and day 25 as chronic colitis (significant numbers of T and B cells)^30^. The mice were divided into groups of 3–5 mice. In the control group, the animals received fresh water. Fresh DSS solution was prepared daily. Body weight, stool consistency, and posture and fur texture were recorded daily to determine the daily disease activity index (DAI). The DAI scoring was assessed blindly, with a maximum score of 10, as described previously^44^. DAI scoring combined the scoring from weight loss (0–4), stool consistency (0–4), and posture and fur texture (0–2). Briefly, a percentage weight loss score of 0 = no loss, 1 = 1–3% loss, 2 = 3–6% loss, 3 = 6–9% loss, and 4 = greater than 9% loss in body mass. A stool consistency score of 0 = no change, 1 = mild change, 2 = loose stool, 3 = loose stool and rectal bleeding, 4 = diarrhea and rectal bleeding. A fur and posture score 0 = no change, 1 = mild hunched posture, 2 = hunched posture and reduced movement. The mice in the hUCMSCs treatment group were injected via the intraperitoneal route with 2.0 × 10^6^/200 μL hUCMSCs on day 1 after receiving DSS. The mice in the PBS group received only PBS 200 μL injection via the intraperitoneal route on day 1 after receiving DSS.

### Histology

The mice in each group were anesthetized and sacrificed on days 3, 8, and 25. Colons were removed, and the distal 3 cm of colon was fixed in 10% formalin and stained with hematoxylin and eosin according to standard histological procedures.

Histological quantification was performed in the colon sections by assessing a histological score as previously described^45^. The inflammatory score comprises the inflammatory cell infiltration score and the intestinal architecture score, with maximum values of 3 points each. In the evaluation of inflammatory cell infiltration, no inflammatory cell infiltration received a score of 0, mild mucosal infiltration received a score of 1, mucosal and submucosal inflammatory cell infiltration received a score of 2, and inflammatory cell transmural infiltration received a score of 3. In the evaluation of intestinal architecture, an intact epithelium received a score of 0, focal epithelial erosion received a score of 1, widespread epithelial erosion received a score of 2, and extensive ulcerations received a score of 3. The inflammatory score was defined as the sum of the scores for inflammatory cell infiltration and intestinal architecture.

### ***In vivo*** imaging of stem cells stained with near-infrared (NIR) dyes

For cell tracking studies, NIR fluorescent dye, XenoLight DiR (DiIC18[7] or 1,1′-dioctadecyl-tetramethyl indotricarbocyanine iodide) (Caliper Life Sciences, Hopkinton, MA) was used for labeling the hUCMSCs as previously reported^46^. The excitation and emission spectra of DiR is in the near infrared range (excitation, 748 nm and emission, 780 nm). Every 1 × 10^7^ cells were incubated with 10 mL DiR solution (PBS containing 3.5 μg/mL dye and 0.5% ethanol) for 30 min at 37 °C. The labeled cells were washed twice with warm fresh medium by centrifugation at 1500 rpm for 5 min to ensure complete removal of any unbound dye^43^. The mice in each group were anesthetized and sacrificed on days 3, 8, and 25, respectively. hUCMSCs were labeled with DiR and tracked using the whole-body cooled charge-coupled device camera system (IVIS Lumina Series III; PerkinElmer, Waltham MA, USA). The distribution and fluorescence intensity of the DiR-labeled cells were monitored on days 3, 8, and 25. After the mice were sacrificed, the organs including lungs, liver, spleen, kidneys, colon, heart, mesenteric lymph nodes, testes, seminal vesicles, and bladders were placed into the light-tight chamber of the charge-coupled device camera system, and a gray-scale organ surface reference image (digital photography) was taken under weak illumination. Photons emitted from the DiR-labeled cells within the animal organs were quantified using the software program IVIS Lumina Series III software (Perkin Elmer, Waltham MA, USA).

### Cytokines and chemokines

On the days the mice were sacrificed, blood from the venous plexus behind the eyes was collected in a capillary tube. The serum was collected after centrifugation at 6,000 rpm for 10 min and then stored at −80 °C. Serum concentrations of a broad panel of cytokines and chemokines were measured using the ProcartaPlex Mouse Cytokine and Chemokine Panel 1 (26-plex) (Thermo Fisher Scientific, Vienna, Austria) according to the manufacturer’s instructions. The panel included the following proteins: IFN-γ, IL-12p70, IL-13, IL-1β, IL-2, IL-4, IL-5, IL-6, TNF-α, IL-18, IL-10, IL-17A, IL-22, IL-23, IL-27, IL-9, GRO-α, IP-10, MCP-1, MCP-3, MIP-1α, MIP-1β, MIP-2, RANTES, eotaxin, and GM-CSF.

### Statistical analysis

Experimental results are expressed as means ± standard error of the mean (SEM). Nonparametric analyses between two groups were performed using the Mann-Whitney U test. P < 0.05 was considered statistically significant.

## Supplementary information

Supplementary Information

## Data Availability

All data generated or analyzed during this study are included in this published article (and its Supplementary Information files).
