# Supplementary material for: Therapeutic effects of a single injection of human umbilical mesenchymal stem cells on acute and chronic colitis in mice
Source: Sci Rep. 2019 Apr 9;9:5832. doi: 10.1038/s41598-019-41910-x (PMC6456500; doi:10.1038/s41598-019-41910-x)
Supplement: Supplementary file 1 — Supplementary Information [file 41598_2019_41910_MOESM1_ESM.pdf]

Supplementary Information

Therapeutic effects of a single injection of human umbilical mesenchymal stem cells on acute and chronic colitis in mice

Yu-Lung Chang[1,2,3,7], Huei-Yu Lo[4,7,8], Shun-Ping Cheng[4,7], Kuo-Ting Chang[7], Xiu-Fang Lin[2], Sheng-Pien Lee[1], Ming-Fa Hsieh [2*], Chin-Kan Chan[5,6,7*]

[1] Department of Urology, Taoyuan General Hospital, Ministry of Health and Welfare, Taoyuan, Taiwan

[2] Department of Biomedical Engineering, College of Engineering, Chung Yuan Christian University, Chung Li, Taiwan

[3] Department of Urology, School of Medicine, National Yang-Ming University, Taipei, Taiwan

[4] Department of Rehabilitation, Taoyuan General Hospital, Ministry of Health and Welfare, Taoyuan, Taiwan

[5] Department of Pediatrics, Taoyuan General Hospital, Ministry of Health and Welfare, Taoyuan, Taiwan

[6] Department of Biotechnology, School of Health Technology, Ming Chuan University, Taoyuan, Taiwan

[7]Translational Medicine Center, Taoyuan General Hospital, Ministry of Health and Welfare, Taoyuan, Taiwan

[8]Department of Chemistry, Chung Yuan Christian University, Chung Li, Taiwan

Supplementary Table S1

P value of disease activity index (Control group vs. PBS group) (Mann-Whitney U test)

Day	0	1	2	3	4	5	6
P	0.6531	<0.0001	<0.0001	<0.0001	<0.0001	<0.0001	<0.0001
Day	7	8	9	10	11	12	13
P	<0.0001	<0.0001	0.0003	0.0006	0.0005	0.0005	0.0081
Day	14	15	16	17	18	19	20
P	0.0038	0.0038	0.0062	0.0034	0.0062	0.0034	0.0073
Day	21	22	23	24	25		
P	0.0062	0.0073	0.0062	0.0056	0.0034		

Supplementary Table S2

P value of disease activity index (Control group vs. hUCMSCs group) (Mann-Whitney U test)

Day	0	1	2	3	4	5	6
P	0.7101	0.0158	<0.0001	<0.0001	<0.0001	<0.0001	0.0001
Day	7	8	9	10	11	12	13
P	<0.0001	<0.0001	0.0005	0.0011	0.0007	0.0016	0.0280
Day	14	15	16	17	18	19	20
P	0.0055	0.0047	0.0209	0.0055	0.0253	0.0055	0.0477
Day	21	22	23	24	25		
P	0.0209	0.0477	0.0253	0.0253	0.0047		

Supplementary Table S3

P value of disease activity index (PBS group vs. hUCMSCs group) (Mann-Whitney U test)

Day	0	1	2	3	4	5	6
P	0.9481	0.0002	0.0326	0.0117	0.0349	0.0362	0.0001
Day	7	8	9	10	11	12	13
P	0.0010	0.0007	0.0291	0.0286	0.0023	0.0025	0.0339
Day	14	15	16	17	18	19	20
P	0.0308	0.0262	0.0308	0.0396	0.0262	0.0262	0.0262

Day	21	22	23	24	25		
P	0.0456	0.0396	0.0262	0.0219	0.0219		

Supplementary methods

Immunohistochemistry staining for the detection of hUCMSCs

Paraffin embedded organs of hUCMSCs groups including lungs, liver, spleen, kidneys, colon, heart, testes, seminal vesicles, and bladder were cut into 5 μm thick sections, placed on poly-L-lysine coated slides, deparaffinized in a 70 °C oven for 20 minutes and in xylene for 20 minutes and rehydrated with graded alcohol. Tissue sections were incubated in Epitope Retrieval Solution pH 6 (Leica: RE7113-CE, Leica Microsystems (SEA) Pte Ltd., Harbourfront Centre Singapore, Singapore) for 15 min in an electronic pressure cooker (Panasonic SR-P37, Panasonic Corporation, Osaka, Japan) for antigen retrieval. Further steps were performed according to the manufacturer's instructions. Tissue sections were incubated with mouse anti-human nuclei primary antibody (merck Millipore: MAB1281, Merck KGaA, Darmstadt, Germany) at 4°C overnight in a humidified chamber. The sections were then incubated with the indicated goat anti-mouse secondary antibody (KPL, 5220-0286, SeraCare Life Sciences Inc., Milford MA, USA) for 30 min at room temperature. Horseradish peroxidase activity was detected by 3, 3′diaminobenzidine (DAB), which served as a

chromogen (DAB; BiotnA Biotech, Kaohsiung, Taiwan), for 15 minutes. All the steps were washed 3 times in PBS for 5 minutes each. The sections were finally counterstained with hematoxylin, dehydrated with alcohol, cleared in xylene and mounted with Neo-Mount. Negative controls were also performed without applying the primary antibody.

Supplementary Figures

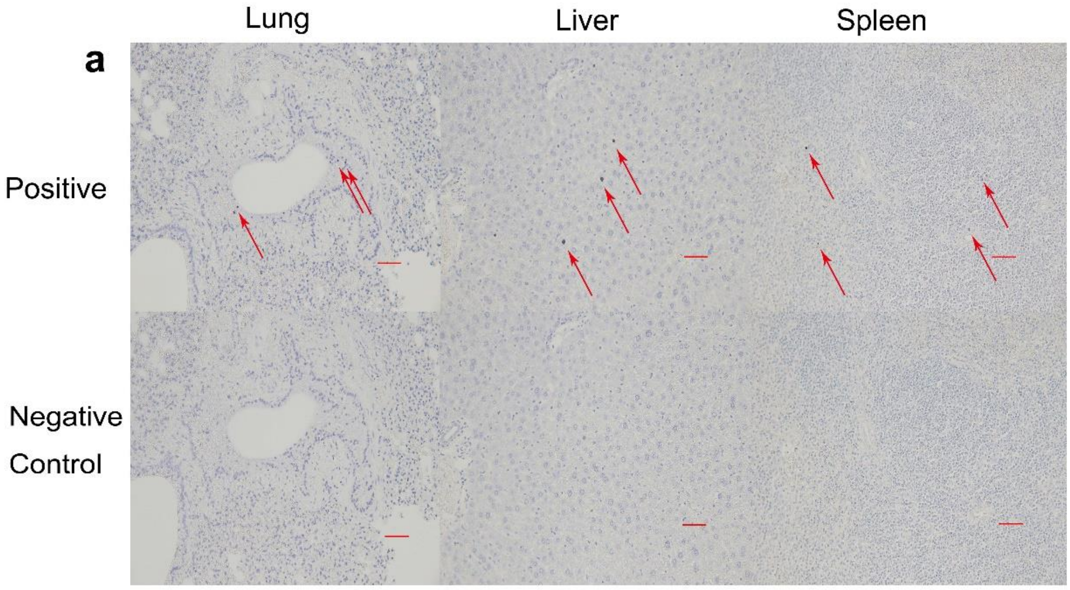

b

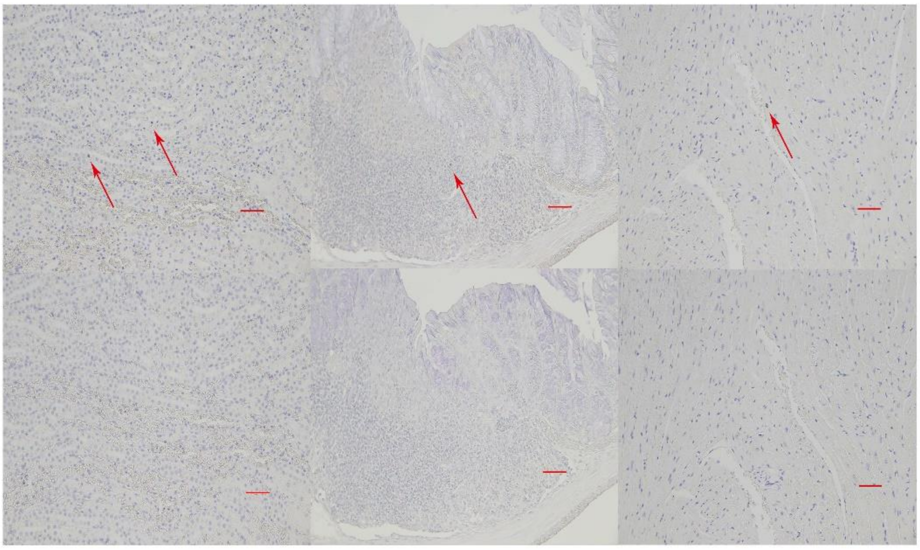
Kidney
Colon
Heart
Positive
Negative Control

c

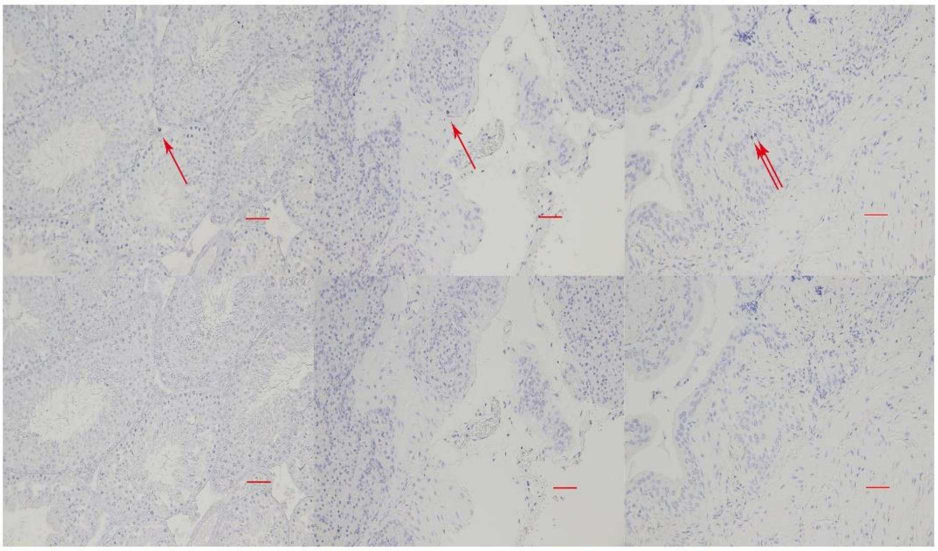
Testis
Seminal vesicle
Bladder
Positive
Negative Control

Supplementary Fig S1 Immunohistochemistry staining for hUCMSCs in mouse organs on day 3. The hUCMSCs were stained with a mouse anti-human nuclei monoclonal antibody and a goat anti-mouse secondary antibody (Positive). Negative control was performed without applying primary antibody. The red arrows point out the hUCMSCs. Original magnification × 200. Scale bar is 50 µm.

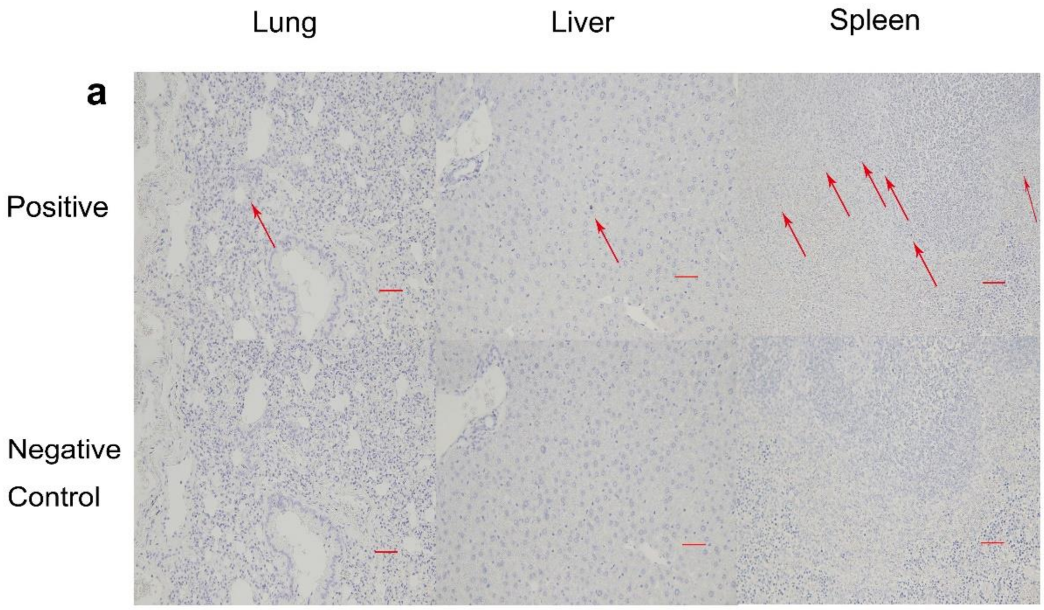

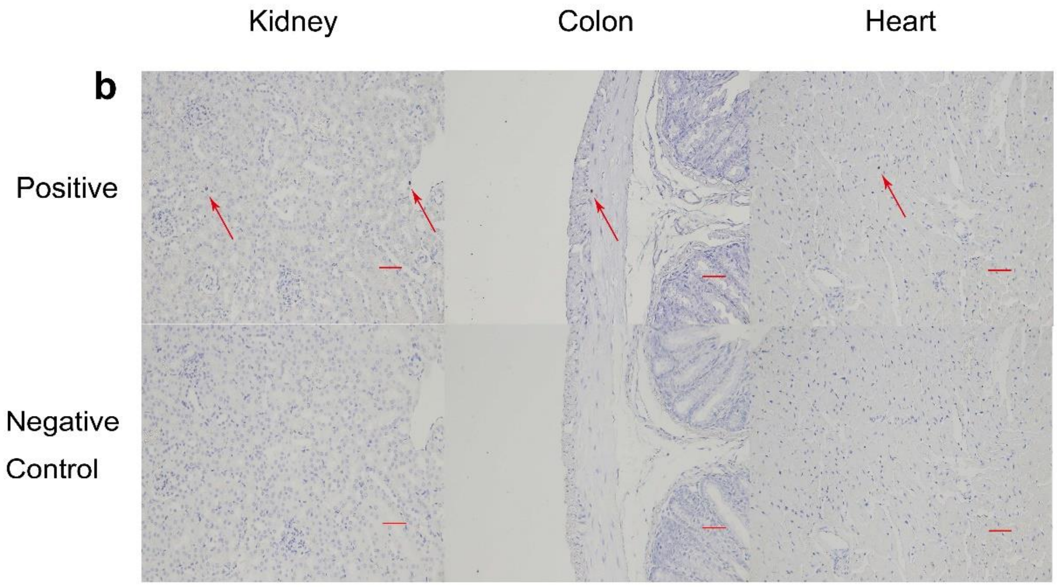
b
Kidney
Colon
Heart
Positive
Negative
Control

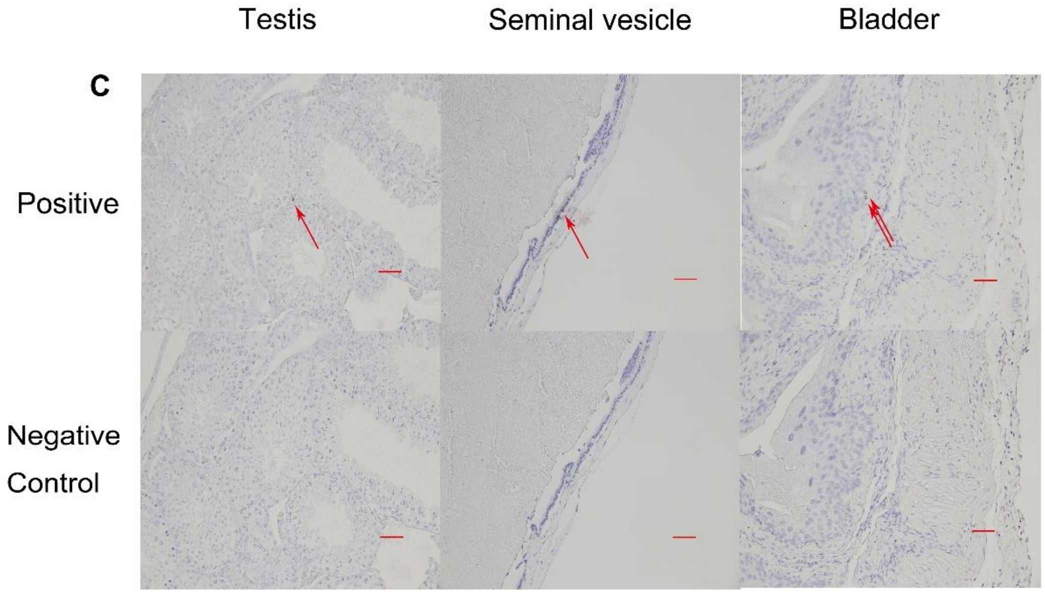
c
Testis
Seminal vesicle
Bladder
Positive
Negative
Control

Supplementary Fig S2 Immunohistochemistry staining for hUCMSCs in mouse organs on day 8. The hUCMSCs were stained with a mouse anti-human nuclei monoclonal antibody and a goat anti-mouse secondary antibody (Positive). Negative control was performed without applying primary antibody. The red arrows point out the hUCMSCs. Original magnification × 200. Scale bar is 50 μm.

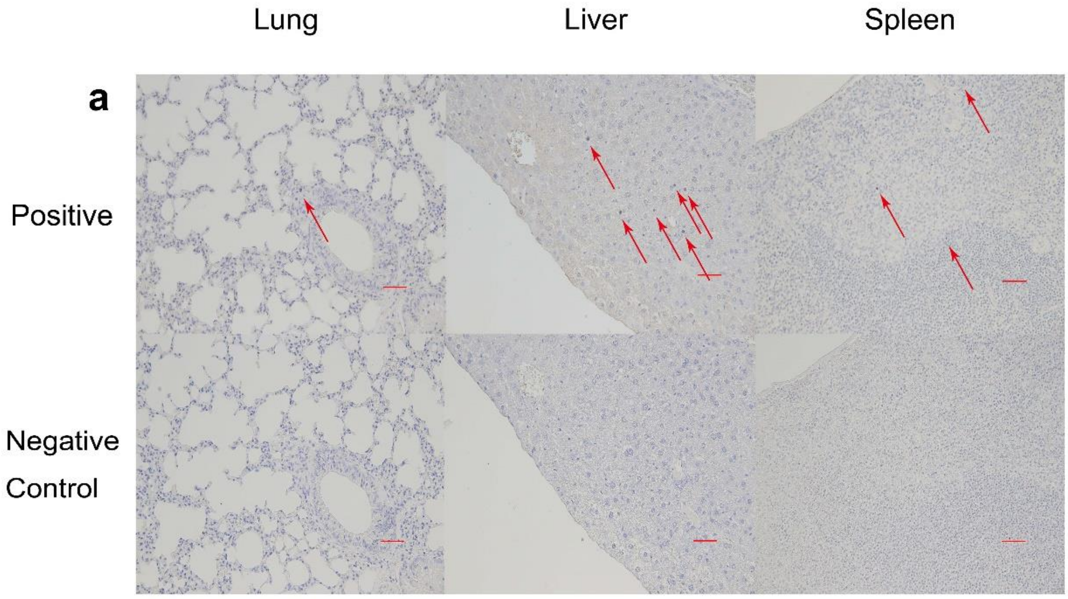

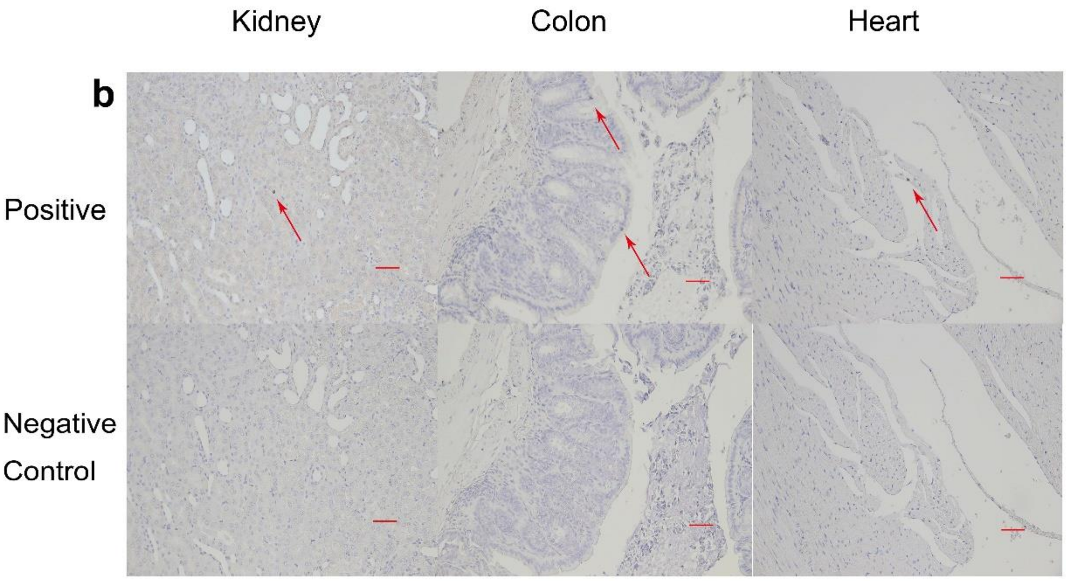
Kidney
Colon
Heart
b
Positive
Negative
Control

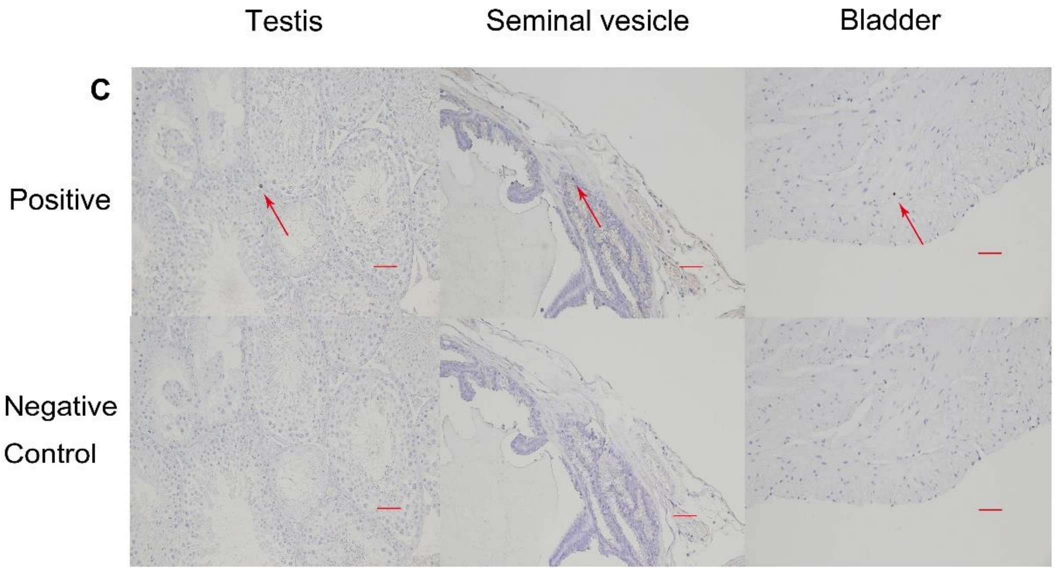
Testis
Seminal vesicle
Bladder
c
Positive
Negative
Control

Supplementary Fig S3 Immunohistochemistry staining for hUCMSCs in mouse organs on day 25. The hUCMSCs were stained with a mouse anti-human nuclei monoclonal antibody and a goat anti-mouse secondary antibody (Positive). Negative control was performed without applying primary antibody. The red arrows point out the hUCMSCs. Original magnification × 200. Scale bar is 50 μm.
